# The 50-Year Journey of Lung Cancer Screening: A Narrative Review

**DOI:** 10.7759/cureus.29381

**Published:** 2022-09-20

**Authors:** Aneri Parekh, Kunal Deokar, Mrinalini Verma, Sanjay Singhal, Madan Lal Bhatt, CDS Katoch

**Affiliations:** 1 Pulmonary Medicine, All India Institute of Medical Sciences, Rajkot, Rajkot, IND; 2 Radiation Oncology, King George's Medical University, Lucknow, IND

**Keywords:** chest radiograph, screening, ldct, early detection, lung cancer

## Abstract

Early diagnosis and treatment are associated with better outcomes in oncology. We reviewed the existing literature using the search terms “low dose computed tomography” and “lung cancer screening” for systematic reviews, metanalyses, and randomized as well as non-randomized clinical trials in PubMed from January 1, 1963 to April 30, 2022. The studies were heterogeneous and included people with different age groups, smoking histories, and other specific risk scores for lung cancer screening. Based on the available evidence, almost all the guidelines recommend screening for lung cancer by annual low dose CT (LDCT) in populations over 50 to 55 years of age, who are either current smokers or have left smoking less than 15 years back with more than 20 to 30 pack-years of smoking. “LDCT screening” can reduce lung cancer mortality if carried out judiciously in countries with adequate resources and infrastructure.

## Introduction and background

Globally, in 2020, there were 2,206,771 new cases of lung cancer [1]. It is now the second most commonly diagnosed cancer worldwide, after breast cancer, but still, it is the most common cause of mortality due to cancer worldwide, accounting for 1,796,144 deaths in 2020, which is 18% of total cancer-related deaths [1]. In India, lung cancer in 2020 accounted for 72,510 (5.5%) of the total incident cancer cases [2]. In India, lung cancer is the fourth most commonly diagnosed, after breast, oral cavity, and cervical cancer. It is the fourth leading cause of mortality among cancers, accounting for 66,279 deaths. It accounted for 7.8% of cancer-related deaths in 2020 [2]. According to the Indian Council of Medical Research (ICMR) report 2020, 44% of cases in male and 47.6% of cases in female population were diagnosed with distant metastasis (M1 disease), 37% of cases in male and 29.8% of cases in female population were diagnosed with locally advanced stage, and only 13.9% and 17% cases were diagnosed at a localized stage among males and females, respectively [3]. In half of the cases, this late detection at the metastatic stage (M1) is the culprit of high mortality. As survival time decreases significantly with the disease stage progression, five-year survival is 45%-50% for a localized early clinical stage I, 10% for stage III, and only 2% for metastatic stage IV disease [4].

Only stage I and limited stage II lung cancers are amenable to curative surgical resection [5]. This further highlights the significance of secondary screening to diagnose patients at an early stage so that curative treatment can be offered to achieve maximum overall survival.

The idea of screening for lung cancer dates back to 1963 when Saccomano et al. [6] demonstrated a technique to detect malignant cells in sputum. Based on this, several trials have been done to evaluate the role of sputum and chest x-rays in lung cancer screening [7-14]. However, most of these studies failed to demonstrate a beneficial effect on mortality in the screened population [7-14]. With the advent of low dose computed tomography (LDCT) and its role in the early detection of lung cancer, there is an upsurge of various randomized controlled trials (RCTs) and feasibility studies of LDCT in lung cancer screening in multiple parts of the world. Here, we reviewed all the available documented evidence in the literature.

## Review

Material and Methods

We searched PubMed using the keywords “low dose computed tomography” and “lung cancer screening” published between January 1, 1963 and April 30, 2022. We also used the search engine Google Scholar and scanned through the references of the selected articles to find additional studies. We included systematic reviews with meta-analyses, RCTs, and controlled clinical trials in our review. We also reviewed the guidelines for lung cancer screening given by various professional societies. Only articles published in English were included. To minimize bias, the search was carried out independently by two authors, AP and SS. A third author KD resolved any disagreement.

Results

Single-Arm Studies

It is evident from various single-arm studies that lung cancer can be detected at an early stage with a computed tomography (CT) scan than a chest radiograph [15-17]. The Early Lung Cancer Action Project (ELCAP) showed that early-stage lung cancers (stage-I) were diagnosed six times more frequently on LDCT than on chest radiographs (2.3% [1.5-3.3] versus 0.4% [0.1-0.9]) [15]. Another study by Sone et al. [16] showed a 10-fold increase in the probability of lung cancer detection on mobile CT screening compared to chest radiographs. Despite detecting early lung cancer, analysis of two single-arm cohort studies gave contradictory results on the prevention of lung cancer mortality [15,18]. The first analysis showed that 85% were detected with stage-I lung cancer with an estimated 10-year survival rate of 88% [15]. This analysis concluded that the annual CT screening could prevent 80% of lung cancer deaths [15]. In contrast, another analysis concluded that LDCT might increase lung cancer diagnosis at an early stage and surgical resection, but without reducing advanced lung cancer and lung cancer-related death [18]. Based on the findings of baseline and repeat screening with LDCT of the chest among 3,642 smokers, Pittsburg Lung Screening Study (PLuSS) also concluded that LDCT leads to the detection of early-stage lung cancer at the cost of many diagnostic follow-up procedures, including major thoracic surgery with the non-cancer outcome [19]. These studies ultimately led to the genesis of RCTs for lung cancer screening.

*RCTs and Feasibility Studies Around the World* 

USA: Lung screening study (LSS), a pilot study assessing the feasibility of a large RCT, showed high compliance with screening by LDCT or chest radiograph with minimal cross-over, hence paving the way for the National Lung Screening Trial (NLST) [20]. The NLST was a multicenter RCT that enrolled 53,454 participants of age 55-74 years old with a ≥ 30 pack-years smoking history, either current smokers or who had quit smoking within 15 years [21]. After randomization, 26,722 participants were assigned to the LDCT screening group and 26,732 to the chest radiography group. The participants underwent three screenings with either LDCT or a single view posteroanterior chest radiograph annually, with the first screening just after randomization. The incidence of lung cancer was higher in the LDCT group than in the chest radiography group (645 versus 572 per 100,000 person-years; rate ratio 1.13; 95%CI: 1.03-1.23). The mortality was lower in the LDCT group than in the chest radiography group (247 versus 309 per 100,000 person-years), with a relative reduction in mortality of 20.0% (95% CI, 6.8-26.7; P=0.004). Screening with LDCT also significantly reduced all-cause mortality by 6.7% (95% CI, 1.2-13.6; P=0.02). Even extended follow-up of the NLST showed a sustained reduction in mortality due to lung cancer in the LDCT arm [22]. The number needed to screen to prevent one lung cancer death (NNS) was 303 in extended follow-up, similar to the original reported NNS of 320 [22]. A cohort study as a secondary analysis of NLST data with the inclusion of more black individuals showed a greater mortality reduction due to lung cancer (black individuals: Hazard ratio [HR] 0.82; 95% CI: 0.72-0.92; versus the whole NLST cohort: HR 0.84; 95% CI: 0.76-0.96), hence stressing the need of access to lung cancer screening for black populations with habits of smoking [23].

After several years of LSS study completion, an analysis was performed by linking to the National Death Index (NDI) to determine their long-term mortality [24]. After a median follow-up of 5.2 years, the lung cancer-specific mortality was 3.84 and 3.10 per 1,000 person-years with a risk ratio of 1.24 (95% confidence interval [CI]: 0.74-2.08) in the LDCT group and chest radiograph group, respectively [24].

Italy: In Italy, three small RCTs were conducted to assess the impact of screening with LDCT on the reduction of mortality.

The Detection And screening of early lung cancer with Novel imaging TEchnology (DANTE) trial in 2001 enrolled 1,264 subjects in the LDCT arm and 1,186 in the control arm of the age 60-74 years with a smoking history of at least ≥ 20 pack-years; either current smokers or who have quit within last 10 years [25]. All subjects had undergone chest radiographs and three-day sputum cytology as a baseline, regardless of their allocation. Subjects of the LDCT arm, besides baseline LDCT, also received four rounds of annual screening along with a clinical review. In contrast, the control subjects received a concise clinical review only. In the LDCT arm, 3.71% (47/104 cases) were detected to have stage-I disease, significantly higher than the control group (1.35%, 16/72 cases; P=0.0002). The lung cancer-specific mortality rate was 543 per 100000 patient-years in the LDCT arm, similar to 544 per 100,000 patient-years in the control arm (hazard ratio [HR], 0.933; 95% CI 0.688-1.433), and the all-cause mortality rate was 1,655 per 100,000 patient-years in the LDCT arm compared to 1742 per 100000 patient-years in the control arm (hazard ratio [HR], 0.947; 95% CI 0.769-1.165).

The ITALUNG Lung Cancer screening trial launched in 2004 enrolled 3,206 subjects; aged 55 to 69 years with at least ≥ 20 pack-year smoking history; current or former smokers who have quit smoking within the last 10 years [26]. The participants were randomized to receive LDCT screening annually for four years (n= 1,613) versus usual care (n= 1,593). There was no significant difference in the incidence of lung cancer between the two groups (67 versus 71; RR 0.93; 95% CI 0.67-1.3). However, there was a significantly increased detection of Stage I cancer in the screening group compared to the control group (24 [36%] versus 8 [11%]; p < 0.001). Consequently, the proportion of patients receiving surgical treatment was also significantly higher in the screening group compared to the control group (52% versus 28%; p=0.003). The trial observed a non-significant 30% reduction in lung cancer-specific mortality (RR 0.70; 95% CI: 0.47-1.03; p=0.07) and 17% reduction in all-cause mortality (RR 0.83; 95% CI: 0.67-1.03; p=0.08) after a median follow-up of 9.3 years. Despite lacking statistical significance, this trial shows a consistent temporal relationship as a significant 46% reduction in lung cancer-specific mortality in the post-screening period (RR=0.54; p=0.01).

The Multicentric Italian Lung Detection Study (MILD) in 2005 enrolled 4,099 subjects aged 49-75 years with a ≥ 20 pack-year smoking history who were current smokers or had quit within the last 10 years, without lung cancer history in the previous five years [27,28]. The participants were randomized to a screening arm (n= 2,376), with further randomization to annual (n=1,190) and biennial (n=1,186 LDCT every two years) screening, or a control arm (n = 1,723) with no intervention. MILD was designed to investigate the efficacy of prolonged LDCT screening beyond five years and evaluate biennial screening compared to annual screening [28]. There was a non-significant increased cumulative incidence of lung cancer in the intervention arm compared to the control arm (431 versus 373 per 100,000 person-years; p=0.84) [28]. The detection of stage I lung cancer was also significantly higher in the intervention arm as compared to the control arm (49 versus 13; p=0.0004), and the resection rate was also higher in the intervention arm in comparison to the control arm (64 versus 16; p < 0.0001) [28]. The 10-year cumulative risk of lung cancer-specific mortality was 1.7% in the intervention arm compared to 2.5 % in the control arm, with a significant 39% risk reduction by LDCT (HR 0.61; 95% CI:5-61; p=0.02) [28]. The 10-year cumulative risk of overall mortality was 5.8% in the intervention arm compared to 6.5% in the control arm, with a 20% non-significant risk reduction by LDCT (HR 0.80; 95%CI: 0.62-1.03; log-rank p=0.07) [28]. The landmark analysis beyond five years (excluding lung cancers and death in the first five years) shows a significant 32% risk reduction in overall mortality (HR 0.68; 95%CI: 0.49-0.94, p=0.01) and a 58% risk reduction in lung cancer-specific mortality (HR 0.42; 95%CI: 0.22-0.79, p=0.0037) by LDCT as compared to no screen [28]. Thus, as evident from the MILD trial, screening beyond five years can enhance its effect. Algorithm of more extended screening (two years intervals) after a negative baseline LDCT and annual in case of indeterminate LDCT in the biennial arm detected similar stage-I lung cancer, surgical resections, and interval lung cancers, but with a reduced cost and radiation exposure [29]. Overall mortality (HR: 0.80, 95% CI: 0.57-1.12) and lung cancer-specific mortality (HR: 1.10, 95% CI: 0.59-2.05) were also similar in the biennial LDCT arm as compared to the annual LDCT arm at 10 years [30].

To overcome the small sample sizes in the European trials, pooled analysis of two Italian RCTs (DANTE and MILD) was conducted for 3,640 participants in the LDCT arm versus 2,909 participants in the control arm [31]. After a median follow-up of 8.2 years, 192 lung cancer cases were detected in the LDCT arm compared to 105 lung cancer cases in the control arm, with half of the LDCT arm cases and 21% of the control arm cases having early-stage cancer. This pooled analysis showed a non-significant reduction of 11% in overall mortality with LDCT (HR:0.89; 95%CI: 0.74-1.06). HR of lung cancer-specific mortality was 0.83 (95% CI: 0.61-1.12) for the LDCT arm compared to the control arm.

France: Depiscan, a pilot RCT of LDCT versus chest radiograph, showed that the detection of non-calcified nodules was 10 times more often in the LDCT group than in the chest radiograph group. Lung cancer detection was also higher in the LDCT group (2.4%) than in the chest radiograph group (0.3%); however, a higher proportion of advanced lung cancer in the LDCT group was detected [32].

Denmark: The Danish lung cancer screening trial (DLCST) recruited 4104 participants aged 50-70 years with ≥ 20 pack-year smoking history [33]. The screening group detected significantly higher lung cancer than the control group (100 versus 53; p < 0.001). The detection of early-stage cancers (stage I and II) was also more in the screening group compared to the control group (54 versus 10; p < 0.001). There was no difference between lung cancer-specific mortality (39 versus 38; HR 1.03; p=0.888) and all-cause mortality (165 versus 163; HR 0.82; p=0.867) in both the groups. As DLCST was a statistically underpowered study, so a conclusive statement on the efficacy of LDCT in lung cancer screening is not possible.

Germany: The German Lung Cancer Screening Intervention (LUSI) study recruited 4,052 participants aged 50-69 years with a smoking history of at least 15 cigarettes per day for 25 years, or at least 10 cigarettes per day for at least 30 years, who were current smokers or had quit smoking < 10 years ago [34]. The participants were randomized into a screening arm (n= 2,029) to receive baseline and four annual LDCT screening rounds or a control arm (n=2,023) to receive usual care. On the first detection of nodules in LDCT, any screening round was classified as per the largest diameter. Nodules ≥ 5 mm had undergone earlier follow-up LDCT according to size (5-7mm: 6 months, 8-10 mm: 3 months, ≥ 10 mm: immediate diagnostic work-up). In the screening round, depending upon volume doubling time (VDT), diagnostic workup was recommended (VDT > 600 days: annual screening, VDT: 400-600 days; LDCT after six months, VDT < 400 days; immediate work-up). There was a significant increase in combined diagnosis for early and advanced stage tumors over the screened period in the screening arm compared to the control arm (HR 1.76 [95% CI: 1.17-2.66], p < 0.01). There was a significant increase in the diagnosis of early cancer (stage I) during the active screening period (5 years post-randomization) in the screening arm as compared to the control arm (HR 14.1 [95% CI: 4.37-45.5], p<0.0001). There was a 39% reduction in the detection of advanced cancers in the screening arm by LDCT compared to the control arm (HR 0.61 [95% CI: 0.35-1.07], p=0.083). After two years of randomization, overall lung cancer deaths diverged, resulting in a nonsignificant HR of 0.74 (95% CI: 0.46-1.19; p=0.21). Modeling by sex, however, showed a significant reduction in lung cancer-specific mortality among women (HR 0.31 [95% CI: 0.10-0.96]; p=0.04). There was no significant effect on all-cause mortality (HR 0.99 [95% CI: 0.79-1.29], p=0.95).

Netherland/Belgium: The Nederlands-Leuvens Longkanker Screenings Onderzoek (NELSON) was a population-based randomized controlled trial initiated in Netherlands and Belgium, enrolling 15,792 participants (13,195 males, 2,595 females, and three unknown sex) aged 50 to 74 years, who are current or former smokers [35]. The participants were randomized to 1:1 into the screening group (6,583 male) or the control group (6,612 male). Participants in the screening group had undergone four rounds of LDCT screening at intervals of 1, 2, and 2.5 years, whereas the control group received usual care. Lung nodules detected on LDCT were analyzed with semiautomated software to determine nodule volume. Nodules with volume >500 cubic mm or volume 50-500 cubic mm with VDT < 400 days on repeat CT scan after three months were labelled positive. At 10-year follow-up, a higher cumulative incidence of lung cancer was detected in the male participants of the screening group (5.58 cases/1,000 person-years) than in the control group (4.91 cases/1,000 person-years) with a rate ratio of 1.14 (95% CI: 0.97-1.33). Screening detected substantially more cases of lung cancers 58.6% in stage IA or IB than 13.5% in the control group. At 10 years of follow-up, mortality due to lung cancer in the screened group (156 death; 2.5/1,000 person-years) was lower than in the control group (206 deaths; 3.30/1,000 person-years), with a cumulative rate ratio of lung cancer-specific mortality of 0.76 (95% CI, 0.61-0.94; p=0.01). Subgroup analysis among women revealed a further reduced rate ratio of 0.67 (95% CI: 0.38-1.14) for lung cancer-specific mortality. All-cause mortality was similar in both the groups as this trial was not sufficiently powered to show a possible favorable difference.

United Kingdom: The UK Lung Study (UKLS) enrolled 4,055 participants aged 50-75 years with a risk score > 4.5% according to version 2 of the Liverpool Lung Project risk model (LLPv2), randomizing participants in the ratio of 1:1 to LDCT arm (2,028 patients) or usual care arm (2,027 patients) [36]. The median follow-up was 7.2 years. There was a non-significant increase in the incidence of lung cancer in the screening arm compared to the control arm (86 versus 75; RR: 1.15 [95%CI: 0.8-1.57], p=0.375). The lung cancer-specific mortality was lower in screened group compared to the control group, but the difference was not statistically significant (30 versus 46; RR 0.65 [95%CI: 0.41-1.02], p=0.062). Out of 161 patients diagnosed with lung cancer, the number of deaths was significantly lower in the screening arm compared to the control arm (42 versus 58; RR 0.52 [95%CI: 0.35-0.77], p=0.001). The odds of cancer being diagnosed at a late stage (stage III or IV) were significantly lower in the screening arm than in the control arm (OR 0.14 [95% CI: 0.07-0.32], p< 0.001). Overall, there were significantly fewer late-stage lung cancers in the screening arm compared to the control arm (16 versus 37; RR 0.43 [95% CI: 0.24- 0.77], p=0.005).

The LungSEARCH study enrolled 1568 current/ex-smokers with mild/moderate chronic obstructive pulmonary disease (COPD) [37]. Participants were randomized (1:1) to the screened group undergoing annual screening with sputum cytology and cytometry or the control group with no specific procedure. Participants in the screened group with abnormal sputum results were offered annual LDCT and autofluorescence bronchoscopy (AFB), whereas those with normal sputum reports underwent annual sputum analysis. Among 78 diagnosed lung cancer cases, 54.8% (23 out of 42 cases) in the screened group and 45.2% (14 out of 31 cases with known staging) in the control group were diagnosed with early stage. However, this sequential strategy using sputum analysis in detecting high-risk populations for LDCT failed to improve the efficiency and stage-shift in lung cancer screening.

Australia: Queensland Lung Cancer Screening Study (QLCSS) assessed the feasibility of LDCT screening in Australia after applying the NLST protocol with modifications of age criteria from 55-74 years to 60-74 years and the requirement of minimum lung function (forced expiratory volume in 1 second ≥50% predicted on spirometry) among 256 participants [38]. After a median follow-up of five years (1,825 days), detection of lung cancer was higher in the QLCSS study as compared to the NLST study (121 versus 65 cases per 10,000 person-years), which may be due to the recruitment of an older population, predominantly male population, relatively high smoking pack-years and self-reported exposure of occupational asbestos than NLST. 83.3% of diagnosed lung cancer after LDCT screening were stage I-II which is quite similar to NLST. Despite a small sample size and less robust risk-benefit analysis, this study demonstrates the feasibility of LDCT screening in Australia, consistent with NLST.

Brazil: In the Brazilian Lung Cancer screening trial (BRELT1) [39], 790 participants were included with inclusion criteria similar to the NLST criteria. Despite a significantly high positive baseline-LDCT scans in this study (312 of 790 [39.5%]) in comparison to NLST (7,191 of 26,722 [26%]) (p<0.001), a similar number of lung cancer cases were identified (1.0% versus 1.3%) with the equivalent number of invasive procedures, supporting the effectiveness of LDCT screening even in a country with a high incidence of granulomatous diseases like tuberculosis.

China: In a study by Yang et al. [40], in 6,657 participants from an asymptomatic high-risk Chinese population with both smoking (current or former smokers) and non-smoking related risk factors, higher numbers of lung cancer were detected in the LDCT group (51 patients, 1.5%) in comparison to the control group (10 patients, 0.3%). The detection of stage-I lung cancer was also higher in the screened group (48 patients; 94.1%) than in the control group (two patients; 20%). This trial concluded that LDCT had significantly enhanced the detection of early-stage lung cancer by 74.1% as compared to standard care.

India: Garg et al. [41] showed that the odds of detecting lung cancers in the LDCT group were 7.70 (95% CI: 0.39-151.22) compared to the observation group.

A retrospective study from Mumbai, India, among 350 smokers who were screened with LDCT showed lung nodules in 335 (93%), with Lung-RADS category one nodules in 117 (36%), category 2 in 133 (41%), category 3 in 29 (9%), and category 4 in 46 (14%) of positive scans [42]. Seven of the category four nodules were diagnosed to have lung cancer. This pilot study from India showed that LDCT could pick up lung cancer even in a tuberculosis-endemic country like ours.

Meta-analysis

Seven published meta-analyses (Table 1) demonstrated conclusive evidence of a statistically and clinically significant reduction in lung cancer-specific mortality with LDCT screening [36,42-48]. These meta-analyses also showed a slight decrease in all-cause mortality, although not powered sufficiently to give conclusive evidence; however, a small reduction would represent a large number of saved lives if a worldwide lung cancer screening strategy were implemented in the context of the second most commonly detected cancer having second highest mortality rates.

**Table 1:** Summary of all meta-analyses for lung cancer screening

S.N.	Author, year	No. of studies	Trials included (S.N. in table 1)	LDCT arm	Control	Total Lung cancer	Early-stage lung cancer	Lung cancer-specific mortality	All-cause-mortality
1	Fu et al. 2016 [43]	Nine RCTs	1-5, 7,8,11,12	43753	43144	OR 1.31 (95% CI: 1.20-1.43)	OR 2.15 (95% CI: 1.88-2.47)	OR 0.84 (95% CI: 0.74-0.96)	OR 0.96 (95% CI: 0.90-1.02)
2	Huang et al. 2019 [44]	Nine RCTs	1-9	97244 participants		NR	RR 2.08 (95% CI: 1.43-3.03)	RR 0.83 (95% CI: 0.76-0.90)	RR 0.95 (95% CI: 0.90-1.00)
3	Tang et al. 2019 [45]	Nine RCTs	1-6, 8,9,12	38357	37563	RR 1.58 (95% CI: 1.25-1.99, p<0.001)	RR 3.45 (95% CI: 2.08-5.72, p=0.001)	RR 0.84 (95% CI: 0.75-0.95, p=0.004)	RR 1.26 (95% CI: 0.89-1.78, p=0.193)
4	Ebell et al. 2020 [46]	Nine RCTs	1-8 (9 excluded due to high risk of bias)	90475 participants		Cumulative incidence ratio: 1.21 (95% CI: 1.06-1.37)	NR	RR 0.81 (95% CI: 0.74-0.89)	RR 0.96 (95% CI: 0.92-1.01)
5	Sadate et al. 2020 [47]	Seven RCTs	2-8	84558 participants		NR	NR	Relative reduction of 17%, RR: 0.83 (95% CI: 0.76-0.91)	Relative reduction of 4%, RR: 0.96 (95% CI: 0.92-1.0)
6	Hoffman et al. 2020 [48]	Nine RCTs	1,2,4-9	96559 participants		NR	RR 2.93 (95% CI: 2.16-3.98)	RR 0.84 (95% CI: 0.75-0.93)	RR 0.96 (95% CI: 0.91-1.01)
7	Field et al. 2021 [36]	Nine RCTs	1-8, 10	94834 participants			NR	RR 0.84 (95% CI: 0.76-0.92)	RR 0.97 (95% CI: 0.94-1.00)

Guidelines by Various Scientific Societies

Almost all the guidelines recommend screening for lung cancer by annual LDCT in elderly over 50 to 55 years of age who are either current smokers or have left smoking less than 15 years back with more than 20 to 30 pack-years of smoking (Table 2) [49-54].

**Table 2:** Recommendations for lung cancer screening *Presence of one additional risk factor # 20pack-year smoking history if there is an additional cumulative risk of developing lung cancer of ≥5% over the following five years. NCCN - National Cancer Comprehensive Network; USPTF - United States Preventive Services Taskforce; AATS - American Association for Thoracic Surgery; ACS - American Cancer Society; ACCP - American College of Chest Physicians; ERS - European Respiratory Society

Organization	Age	Smoking history pack-years	Smoking cessation	Modality
NCCN Group I [49]	55-74 years	≥ 30 pack-years	< 15 years	Annual LDCT
NCCN Group II [49]	≥ 50 years	≥ 20 pack-years*	--	
USPTF [50]	50-80 years	≥ 20 pack-years	< 15 years	Annual LDCT
AATS [51]	55-79 years	≥ 30 pack-years^#^	--	Annual LDCT
ACS [52]	55-74 years	≥ 30 pack-years	< 15 years	Annual LDCT, Smoking cessation
ACCP [53]	55-77 years	≥ 30 pack-year	< 15years	Annual LDCT
ERS [54]	55-80 years	≥ 30 pack-years	< 15 years	Annual LDCT

All these recommendations are from developed countries like the USA, Europe, and the UK, where the required healthcare infrastructure and trained personnel are readily available with a 10%-16% GDP proportion invested in medical health [55], and people being screened are aware and motivated to pursue follow-up investigations if needed, and specialized centers in managing lung cancers are also readily available.

Compared to the developed countries, lung cancer screening with LDCT is much lower in Asian populations due to various factors. Firstly, 90% of lung cancer patients in the USA had a smoking history compared to 60%-70% of lung cancer patients in Asia [56]. Secondly, certain environmental factors, such as indoor coal burning, environmental tobacco smoke, and cooking oil vapors, could impact the occurrence of lung cancer in developing countries [56]. So, the definition of a high-risk population may differ in these countries compared to developed countries, and these guidelines probably need modifications to be validated in the developing country's populations. Therefore, new criteria for high-risk populations must be defined for the maximum risk-benefit ratio for Asian countries.

Moreover, screening programs should be undertaken only when their effectiveness has been proven locally, along with adequate resources to cover the target population and facilities to confirm the diagnosis and ensure treatment further to convert early detection into survival outcomes. In developing countries, surgical treatments such as thoracotomy and video-assisted thoracoscopy system (VATS) are available only at referral centers that are already overburdened. So, early diagnosis with LDCT will further add to the burden on these healthcare facilities. A patient rendered positive with screening has to undergo a series of procedures for diagnosis, such as CT/USG (Ultrasound Guided) guided transthoracic needle biopsy, thoracoscopy, or bronchoscopy. These facilities are also available at only a few big referral hospitals. Another issue is the cost of treatment and diagnostic procedures. Therefore, the need of the hour is to strengthen the country’s healthcare system by increasing gross domestic product (GDP) expenditure in the medical system to cater to the additional load before implementing any screening program.

Recent and Ongoing Trials

With the identification of potential biomarkers for lung cancer screening, a RCTbegan in Scotland, the Early Diagnosis of Lung Cancer Scotland (ECLS) trial, comparing the use of early CDT-lung test (early cancer detection lung test) in adjunct with LDCT compared to LDCT alone, demonstrating increased detection of early-stage lung cancers in the biomarker group compared to control group [57]. A similar RCT began in Colorado, United States (NCT01700257), reaching early CDT-lung biomarker test with LDCT versus LDCT alone in lung cancer screening; the results are yet to be published [58]. A prospective cohort study began recruitment in March 2006 at New York University, United States (NCT00301119), to assess the role of biomarkers in lung cancer screening, and results are expected to be published by May 2030 [59].

Future directions

More studies are needed to elucidate the role of biomarkers and risk prediction models to improve the sensitivity and reduce the cost of LDCT screening. As trained human resources are scarce, the role of AI in improving risk prediction and nodule detection needs further studies. Though a few studies have shown that the decrease in the specificity of LDCT in TB endemic countries has no clinical impact, more RCTs are needed to confirm these findings. Also, before the implementation of any screening program, especially in developing countries, cost-effectiveness analysis definitely plays an important role. Modelling studies to determine the cost-effectiveness of lung cancer screening programs alone and combined with smoking cessation programs are needed.

## Conclusions

Single arm and pilot studies showed that LDCT increases lung cancer diagnosis, confirmed by RCTs and subsequent meta-analyses. Based on this evidence, almost all the guidelines recommend screening for lung cancer by annual LDCT in elderly more than 50 to 55 years of age who are either current smokers or have left smoking less than 15 years back with more than 20 to 30 pack-years of smoking. However, for a successful screening program, the required healthcare infrastructure and trained resources should be readily available, people being screened should be aware and motivated to pursue follow-up investigations if needed, and centers specializing in the management of lung cancers should be readily available. Also, the financial implications of the screening should be taken into account. Therefore, the country’s healthcare system must be strengthened to cater additional load before implementing any screening program.
